# Dependence of Connectivity on the Logarithm of Geometric Distance in Brain Networks

**DOI:** 10.3389/fphys.2020.611125

**Published:** 2021-01-28

**Authors:** Michele Castelluzzo, Alessio Perinelli, Davide Tabarelli, Leonardo Ricci

**Affiliations:** ^1^Department of Physics, University of Trento, Trento, Italy; ^2^CIMeC, Center for Mind/Brain Sciences, University of Trento, Rovereto, Italy

**Keywords:** brain network, time series, cross correlation, magnetoencephalography, network structure, connectivity

## Abstract

Physical connections between nodes in a complex network are constrained by limiting factors, such as the cost of establishing links and maintaining them, which can hinder network capability in terms of signal propagation speed and processing power. Trade-off mechanisms between cost constraints and performance requirements are reflected in the topology of a network and, ultimately, on the dependence of connectivity on geometric distance. This issue, though rarely addressed, is crucial in neuroscience, where physical links between brain regions are associated with a metabolic cost. In this work we investigate brain connectivity—estimated by means of a recently developed method that evaluates time scales of cross-correlation observability—and its dependence on geometric distance by analyzing resting state magnetoencephalographic recordings collected from a large set of healthy subjects. We identify three regimes of distance each showing a specific behavior of connectivity. This identification makes up a new tool to study the mechanisms underlying network formation and sustainment, with possible applications to the investigation of neuroscientific issues, such as aging and neurodegenerative diseases.

## 1. Introduction

The application of network analysis methods on structural and functional brain connectivity is a widely used tool to investigate the topology of complex brain networks emerging during both resting state and cognitive engagement in a task. This approach led to strong evidence of human brain networks exhibiting a small-world topology (Bullmore and Sporns, 2012; Gastner and Ódor, 2016; Bassett and Bullmore, 2017) and challenged the general assumption of network stationarity (Chang and Glover, 2010; Bassett et al., 2011; Bullmore and Sporns, 2012; Nicol et al., 2012; Brookes et al., 2018) suggesting a dynamic continuous reconfiguration of brain complex networks both in cognitive tasks and at rest. Moreover, small-world topology supports the well-established principle of segregated/integrated information processing (Bullmore and Sporns, 2012). Instantiating and running a functional brain network has a metabolic cost in term of glucose and oxygen consumption required to sustain information processing and circulation. This wiring cost is ultimately related to the distance between communicating brain regions and is supposed to be minimized by the brain, while preserving the crucial computational advantages conferred by network complexity (Bullmore and Sporns, 2012; Gollo et al., 2018). Moreover, an unbalance between wiring cost and complexity has been shown to act as a potential source for neuropsychiatric disorders (Gollo et al., 2018). As a result of the tradeoff between network complexity and wiring cost, brain connectivity and its strength turn out to depend on physical distance between communicating nodes (Bullmore and Sporns, 2012). While actual anatomical link length does not coincide with a straight segment, Euclidean distance appears to be a relevant parameter in the study of brain topology and connectivity (Ghosh et al., 2008; Supekar et al., 2009; Kaiser, 2011; Cabral et al., 2014), making up a lower bound to the real anatomical distance (Avena-Koenisberger et al., 2018). Since the discovery of resting state networks, like the Default Mode Network (Damoiseaux et al., 2006), resting state activity has been extensively used to probe general properties of brain networks. However, despite the relevance of the topic, to our best knowledge only a few studies addressed directly the dependence of connectivity on geometric distance in the brain.

Salvador et al. (2005a,b) analyzed fMRI resting state activity of 12 young healthy adults. They computed partial correlation between 90 cortical and subcortical brain regions, demonstrating for the first time that the intra-hemispheric connectivity was generally related to Euclidean distance by an inverse square law. This result was confirmed by another study (Fair et al., 2009) focused on the development of long distance integrated processing in healthy children and young adults. Authors defined networks from correlation coefficients matrices of resting state fMRI data, confirming that connectivity strength in active links depends on the inverse of the square of Euclidean distance, as found in Salvador et al. (2005a,b). These results were challenged by subsequent studies pointing to different degrees of interplay between distance and connectivity. In Expert et al. (2011) the authors investigated scale invariance in brain networks by analyzing time series from fMRI resting state activity of seven young healthy adults. By computing correlation coefficients between brain areas at different levels of spatial granularity, they found, for short distances, a self-similar behavior of the network, which corresponds to a power-law dependence of correlation on distance in the form of the inverse of the square root of Euclidean distance. Moreover, the self-similar regime, and thus the power-law dependence, is lost for long distances (about > 50 mm). Furthermore, in another paper, it was found that the global mean Euclidean distance of links from a brain network defined by correlating resting state fMRI activity increases when weaker links are included in the network by lowering the threshold that defines the adjacency matrix (Alexander-Bloch et al., 2013). In that work the authors considered results from resting state fMRI activity and found that an exponential curve reasonably fits the cumulative distribution of distances of network links.

Besides results being not consistent, there are also some limitations in the above mentioned studies. On the one hand, using fMRI resting state time series might not reveal the contribution of networks operating at time scales faster than the typical time resolution of magnetic resonance imaging, whose contribution is convoluted with the hemodynamic response. In addition, as pointed out also in Coquelet et al. (2017), confounds might be introduced by the different neurovascular coupling in different healthy and clinical population, or even within the analyzed population. Using a direct electrophysiological measure of brain activity, such as magnetoencephalography (MEG)—characterized by a higher sampling rate can be more efficient in addressing the dependence of connectivity on distance.

In a recent paper by our group (Perinelli et al., 2019), we used a novel method to evaluate connectivity between two brain nodes by means of the time scale, henceforth referred to as “time scale of observability,” in which a correlation is observed. Starting from MEG recordings of cortical activity of healthy subjects in resting state, we obtained a time series for each examined node in the brain and, consequently, a time scale of observability of links. The results hinted at a power-law dependence of the time scale of observability with respect to geometric distance.

In this paper, we improved our previous analysis by using a larger dataset containing MEG recordings from 100 healthy subjects in resting state and, in addition, upgraded analytical tools. The subjects have an age ranging from 18 to 88 years and were chosen to yield an approximately uniform age distribution. A number of 72 nodes was randomly selected on the cerebral cortex and correlation among all possible 42 pairs of nodes was evaluated as a function of pair distance. In addition node pairs were distinguished into inter-hemispheric and intra-hemispheric in order to highlight possible differences in connectivity in cross-hemisphere or intra-hemisphere communication.

We obtained evidence of three different regimes of linear dependence of the time scale of observability on the natural logarithm of geometric distance. This result proves to be statistically significant and makes up an interesting base point for further investigation, aiming, for example, at relating the different regimes to the small-world network supported principle of segregated/integrated information processing. Our novel approach detects communication links between pairs of brain regions by assessing the time scale of observability as the shortest time window during which the two time series have to be observed to significantly detect a correlation (see section 2.2 for a detailed discussion about that). Because typical values are in the range between 0.2 and 30 s, our approach is more efficient than standard correlation analysis of slow fMRI time series in including potential networks that operate at different time scales and provides a more complete metrics for the characterization of the dependence of connectivity strength on distance.

As in all the relevant previous literature about the dependence of connectivity strength on distance, we use here resting state data. While this is a limitation because, in principle, networks with different properties and topologies can emerge in response to stimuli or in executing actions (Bullmore and Sporns, 2012), we believe that a general property of network brain communication, like the dependence of connectivity strength of distance, does not dramatically change whenever subjects are cognitively engaged in a task. Nevertheless, further investigations are required to confirm this property, especially with regards to the validity of the three-regime dependence mentioned above.

The present paper is organized as follows. Section 2 deals with the description of the available dataset and the related preprocessing, a summary of the method for the evaluation of zero-delay cross-correlation, and the analysis of the functional relation between time scale of observability and distance. The outcomes of this analysis are the topic of section 3. Conclusive remarks on the implications of our results are presented in section 4.

## 2. Materials and Methods

### 2.1. Dataset and Pre-processing

Data used in the present work were obtained from the CamCAN repository (available at http://www.mrc-cbu.cam.ac.uk/datasets/camcan/) (Shafto et al., 2014; Taylor et al., 2016). Data collection was conducted in compliance with the Helsinki Declaration and was approved by the University of Cambridge ethics committee (Cambridgeshire 2 Research Ethics Committee—reference: 10/H0308/50) (Shafto et al., 2014).

We selected data from 100 healthy volunteers (49 females, 51 males), with an approximately uniform age distribution ranging from 18 to 88 years. From the data available in the CamCAN repository, we retrieved MEG resting state and empty room recordings together with MRI anatomical scan for each subject. All data were collected and partially preprocessed at the MRC Cognition and Brain Sciences Unit of the University of Cambridge as follows. Individual anatomical MRI images (T1 weighted, 1 mm of resolution) were collected using a 3 T Siemens TIM Trio scanner with a 32-channel head coil. MEG data were recorded using a 306-channel VectorView MEG system (Elekta Neuromag, Helsinki), with 102 magnetometers and 204 orthogonal planar gradiometers, located in a magnetically shielded room. MEG data were natively sampled at 1 kHz with an high pass filter of 0.03 Hz. Resting state recordings were obtained with the participant in seated position and with eyes closed. Temporal Signal Space Separation (correlation threshold 0.98, 10 s sliding window) has been used to reconstruct missing channels, to filter data and to correct continuous head movement (200 ms time window) (Taulu and Kajola, 2005; Taulu et al., 2005; Taulu and Simola, 2006; Shafto et al., 2014). Vertical and horizontal electro-oculogram (EOG) and electrocardiogram were also available from the database.

We further preprocessed and prepared data using FieldTrip (Oostenveld et al., 2011) and custom MATLAB code (The Mathworks, Natick, MA, USA). Continuous resting state MEG data were filtered (0.5–125 Hz band-pass filter; 49–51 Hz notch filter; Butterworth 4-th order two-pass) and resampled at 250 Hz. Identification of physiological artifacts has been performed by using conservative automatic procedures. Muscular activity was detected by filtering data through a 90–110 Hz band-pass filter (Butterworth 9-th order two-pass), converting the time series to z-score and averaging results over channels. Segment having an average z-score higher than 10 were considered as muscular artifacts and removed from the data. Eye related activity and cardiac activity were removed using an automatic procedure based on Independent Component Analysis (ICA) (Hyvarinen et al., 2001). Independent components were computed for magnetometers and gradiometers separately using an extended infomax algorithm (Lee et al., 1999). Thereupon, variance-stabilized correlation coefficients between each component and cardiac/electroocular channels were first evaluated and then transformed to z-scores. Components yielding scores that exceed a threshold, here set to 2, were considered as artifactual and rejected. This identification/rejection procedure was performed twice to ensure that no artifactual components remain within the data. In the following analysis, only data from planar gradiometers are used. MEG empty room data were preprocessed following the same procedure as for human resting-state data.

Individual anatomical MRI scans, which are provided along MEG data, were processed to obtain head models for the solution of the inverse problem. A model of the cortical mantle (20,484 vertices, 3.1 mm of average source spacing) was obtained by segmenting, correcting and extracting the external surface of the gray matter using SPM (Friston, 2007) and the CAT12 SPM toolbox.^1^ Sulci information was extracted and used to compute an interpolation matrix for the projection of the individual source space to a MNI FreeSurfer average common space (5-th order recursive icosahedron, 20,484 vertices) (Fischl et al., 1999). Each vertex in the MNI common space was labeled as belonging to one of the 360 regions of interest defined in a reference atlas by Glasser et al. (2016). The atlas identifies 360 brain regions by combining structural, diffusion, functional and resting state MRI data from 210 healthy young individuals. In addition, surfaces of the enclosing brain and of the scalp, each made of 20,000 vertices, were extracted for forward model solution and co-registration with MEG data, respectively. All extracted surfaces were co-registered to the position of the participant head in the MEG resting state session by aligning anatomical landmarks (nasion, left/right pre-auricular points). The co-registration was further refined by aligning the scalp surface to the points digitized during the MEG acquisition (see Shafto et al., 2014; Taylor et al., 2016) for further details about head digitization during MEG acquisition).

Source activity was reconstructed on each of the 20,484 vertices from MEG data using a Minimum Norm Estimate method (Ilmoniemi and Sarvas, 2019). An orientation free source model was used, thus leading to three time series for each vertex pointing to the x-, y-, and z-coordinates, respectively. Normalized lead fields were obtained using a single shell model (Nolte, 2003), while the covariance matrix was computed from empty room data. A noise normalized MNE kernel matrix for the inversion of sensors data into brain sources was estimated according to Equation (5.39) of Ilmoniemi and Sarvas (2019). Noise covariance matrix was regularized by 10% and the prior source covariance matrix was estimated from the trace of the leadfield matrix as in Hämäläinen (2005). Finally, we reduced the estimated neural activity by considering the norm of the time vector at each vertex so that for each source, a time series corresponding to the norm of the current dipole vector reconstructed at that location is available.

Before proceeding with correlation analysis, data prepared for each subject were visually inspected to ensure no errors in the preparation process. Sensors data were inspected to confirm, given above mentioned automatic thresholds, the correct rejection of artifacts. Extracted surfaces were plotted and checked, together with MEG sensors and projection of individual sulci data on MNI space, to ensure correct co-registration and interpolation. Finally, the CAMCaN repository also provides MEG data with passive audio and visual stimulation. As a further validation, for each subject, sources were reconstructed using these auxiliary sensor data and the computed kernel, and results were visually inspected to check that the corresponding brain activity was correctly localized in auditory and visual cortices.

**Figure 1:**
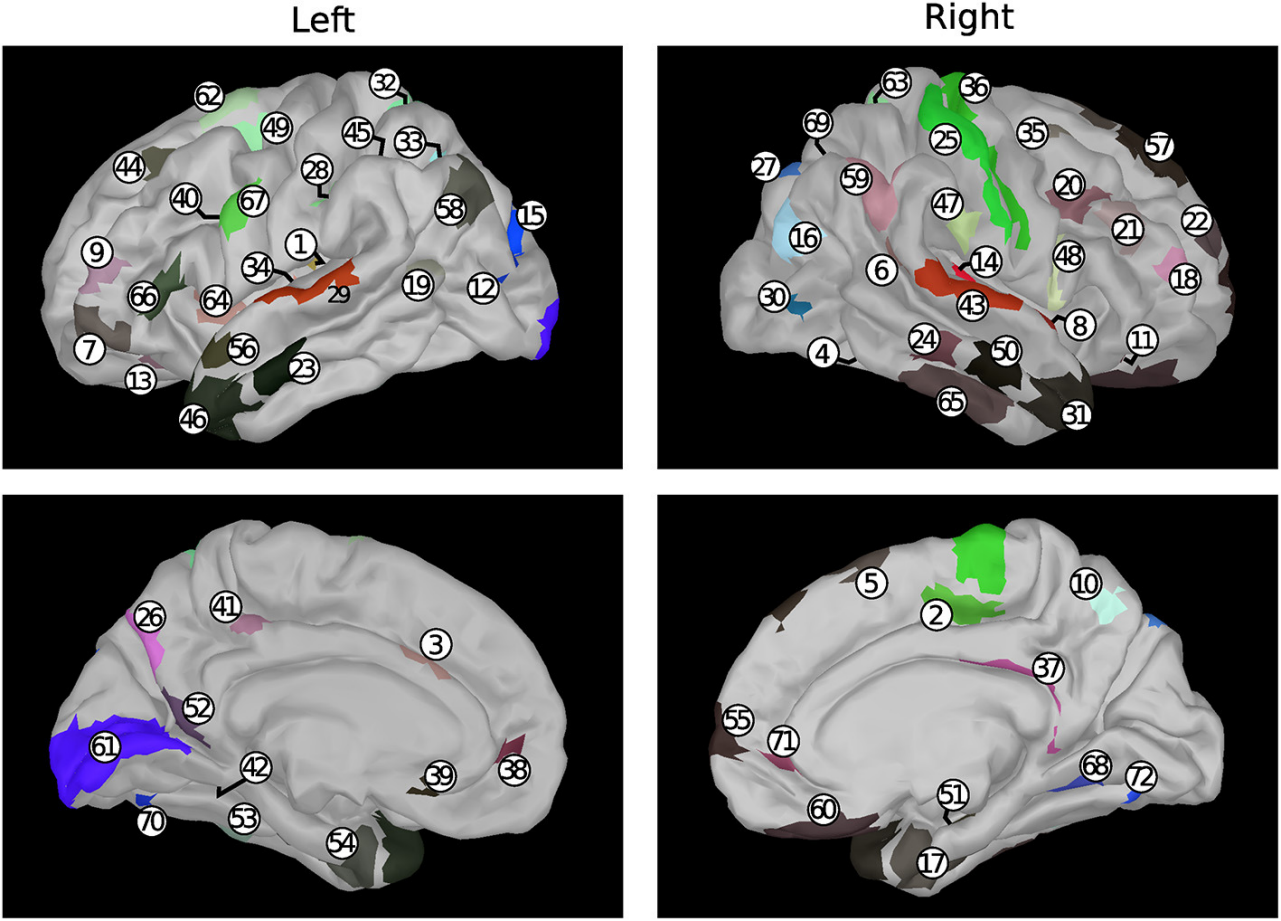
Position, represented on a default anatomy, of the areas listed in Table 1. Colors are related to the functional group of the corresponding area according to the reference atlas (Glasser et al., 2016) considered in the present work.

A set of 72 sources was selected out of the 20,484 available ones provided by the reconstruction process. For each subject, these 72 sources were selected as the closest ones to 72 brain nodes, namely the centroids of 72 brain regions chosen among those defined in the reference atlas (Glasser et al., 2016). Limiting the analysis to 1/5 of the available regions stems from a trade-off between computational cost and adequate coverage of the whole brain. The selection of the 72 brain nodes was carried out randomly and in compliance with a procedure thoroughly described in Perinelli et al. (2019) (section “Dataset and preprocessing” therein). The procedure yields nodes whose pairwise distance is larger than 1 cm, in order to obtain a uniform coverage of the brain cortex. The 72 brain regions extracted through random selection and analyzed in the present work are listed in Table 1, while Figure 1 shows the corresponding anatomical positions.

**Table 1:** Brain areas selected for the present analysis and whose anatomical position is shown in Figure 1.

**Nr**.	**Atlas area (hemisphere)**	**Nr**.	**Atlas area (hemisphere)**	**Nr**.	**Atlas area (hemisphere)**
1	OP2-3 (left)	25	1 (right)	49	6a (left)
2	24dd (right)	26	POS2 (left)	50	TE1a (right)
3	a24pr (left)	27	V6A (right)	51	H (right)
4	TE2p (right)	28	PFt (left)	52	POS1 (left)
5	SFL (right)	29	A4 (left)	53	TE2p (left)
6	STV (right)	30	V4t (right)	54	PeEc (left)
7	a47r (left)	31	TGd (right)	55	10d (right)
8	TA2 (right)	32	7AL (left)	56	STGa (left)
9	a9-46v (left)	33	MIP (left)	57	8BL (right)
10	7Pm (right)	34	PoI1 (left)	58	PGs (left)
11	47m (right)	35	i6-8 (right)	59	PFm (right)
12	V3CD (left)	36	4 (right)	60	OFC (right)
13	13l (left)	37	RSC (right)	61	V1 (left)
14	A1 (right)	38	p32 (left)	62	6ma (left)
15	V3A (left)	39	25 (left)	63	7AL (right)
16	PGp (right)	40	IFJp (left)	64	MI (left)
17	PeEc (right)	41	31a (left)	65	TE2a (right)
18	a9-46v (right)	42	VMV2 (left)	66	45 (left)
19	TPOJ2 (left)	43	A4 (right)	67	6v (left)
20	8C (right)	44	8Ad (left)	68	VMV1 (right)
21	p9-46v (right)	45	IP2 (left)	69	LIPd (right)
22	9a (right)	46	TGd (left)	70	V8 (left)
23	TE1a (left)	47	PFop (right)	71	p32 (right)
24	TE1m (right)	48	6r (right)	72	V8 (right)

*Acronyms pertain to the reference brain atlas defined by Glasser et al. (2016)*.

Muscular artifacts rejection yielded, for each subject, a set of epochs having different duration. Analyzed sequences were extracted by trimming the longest available epoch (having duration *T*_1_) for a given subject, as follows. If *T*_1_ ⩾ 240 s, samples are contemporarily removed from the beginning and from the end of the epoch until the resulting duration is 240 s. If 180 s ⩽ *T*_1_ < 240 s, the same trimming process is carried out until the resulting duration is 180 s. If *T*_1_ < 180 s, the two largest epochs are considered: segments are selected out of these two epochs by means of the same procedure so that their total duration is 180 s. For 10 subjects out of 100, because of insufficient epoch length, the total duration is 160 s. To summarize, analyzed sequences have total duration 240 s for 29 subjects, 180 s for 61 subjects, and 160 s for 10 subjects. The restriction of the analysis to no more than two segments is due to the need of limiting computational cost. Alternatively, one might choose the same minimum duration of 160 s for all subjects. While such a choice would provide a uniform dataset, we rather chose to maximize, whenever possible, the amount of data included in our analysis. It is worth mentioning that the cross-correlation method applied here—and described in section 2.2—yields as outcome a time scale of observability that is independent of the underlying sequence length, provided that such length is much larger than the maximum probed time scale (in this work 30 s).

### 2.2. Link Assessment *via* Zero-Delay Cross-Correlation Analysis

In the present work, the existence of a link between a pair of nodes is assessed out of the corresponding pair of sequences. The assessment is carried out by means of a recently-developed method (Perinelli et al., 2018; Perinelli and Ricci, 2019) based on the evaluation of zero-delay cross-correlation over moving windows having different widths. Cross-correlation is quantified as the sample Pearson correlation coefficient, whereas its significance is estimated through surrogate-based hypothesis testing (Schreiber and Schmitz, 2000). The method is extensively described in Perinelli et al. (2018) and in Perinelli et al. (2019). An implementation of the method is provided in the publicly available NetOnZeroDXC package (Perinelli and Ricci, 2019). The method is here summarized as follows: given a pair of nodes and their corresponding sequences, both of size *N* and sampled with period *T*, the sample Pearson correlation coefficient *r*(*k,w*) is evaluated on a moving window of width *w* and centered at time *t*_*k*_. The window width takes on values given by *mw*_0_, where *w*_0_ is the minimum width and *m* is an integer number between 1 and *M*. The center point of the first window is set to $t_{0}=\frac{Mw_{0}}{2}$, whereas the successive center points are iteratively set according to *t*_*k*+1_ = *t*_*k*_ + *w*_0_ up to the last window that is centered at $t_{K}=NT-\frac{Mw_{0}}{2}$. The center points and, consequently, the number of same-sized windows used to cover the sequences, are the same independently of the window width *w*. The set of center points {*t*_*k*_} are ultimately fixed by the size of the sequences *N* and the choice of *w*_0_ and *M*. In the present work, *w*_0_ was set to 0.2 s and *M* to 150, so that the window width covers a range from 0.2 to 30 s.

These last parameters also set the lower and upper time scale limits within which a link is observable. The upper limit of 30 s is a consequence of the finite duration (several minutes) of the available sequences. The lower limit is instead constrained by the necessity of having a sufficient number of samples in each windows so as to evaluate cross-correlation. In this work, because the sampling period is 4 ms, the minimum window width of 0.2 s corresponds to 50 samples. Smaller time scales could be probed by increasing the sampling rate by one or more orders of magnitude, a possibility hampered by the limitations of current MEG technology.

**Figure 2:**
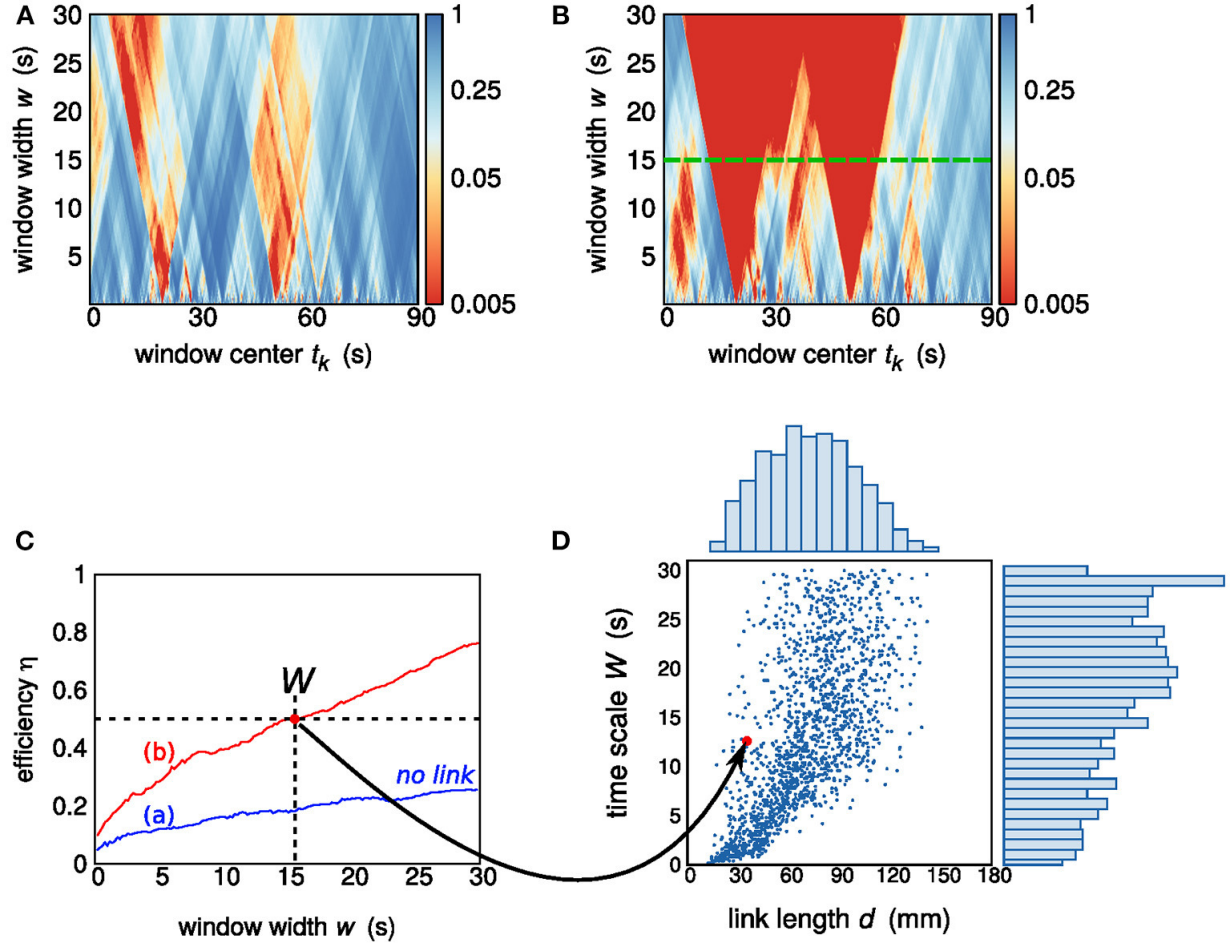
**(A,B)** *P*-value diagrams *p*(*k,w*) of sections of sequences belonging to two pairs of nodes. The diagram in **(A)** corresponds to a pair that is not deemed to be linked, while a link exists in **(B)**. The green dashed line shows the link time scale *W* = 15 s. **(C)** Efficiency η as a function of the window width *w*: the blue and red lines corresponds to the diagrams in **(A,B)**, respectively. The efficiency threshold of 0.5 is reached by the red efficiency curve at *w* = 15 s. **(D)** Scatter plot, for one subject, of the assessed links: each point (link) is identified by the corresponding Euclidean distance *d* and time scale of observability *W*. The pair of nodes exhibiting a link contributes to the scatter plot with a point of abscissa given by the distance *d* between the two nodes (32 mm) and ordinate given by the resulting time scale *W* (15 s). Two histograms, showing the sample marginal probability distributions of *d* and *W*, are also shown.

Given the Pearson correlation coefficient diagram *r*(*k,w*), a *p* value diagram *p*(*k,w*) is obtained by testing a null hypothesis of independence on a number of 200 surrogates for each pair of sequences. Upon setting a *p*-value threshold at 0.05, we defined an *efficiency* function η = η(*w*) which gives the percentage of windows of size *w* showing a significant cross-correlation. Efficiency can be interpreted as an index of how efficient a window width—and therefore the related time scale—is in detecting significant cross-correlations between a pair of sequences (Perinelli et al., 2018). As shown in Figure 2, the efficiency function η(*w*) increases monotonically with *w*. In the present work, a link is deemed to exist if η(*w*) overcomes the efficiency threshold of 0.5. In other words, given a pair of nodes, the existence of a link is acknowledged provided that a window width *w* exists such that the majority of windows detect a significant correlation in the corresponding pair of sequences. The minimum value of *w* at which this crossing occurs is taken as the *time scale of observability* of the corresponding link and is henceforth referred to as *W*. If η(*w*) does not overcome the efficiency threshold for any value of *w*, no link is deemed to exist between the pair of nodes.

While *W* is a measure of time scale of brain dynamics, it is not related to the signal propagation speed within the brain (Fransson, 2005; de Pasquale et al., 2010; Perinelli et al., 2018), which is instead characterized by time scales that are three orders of magnitude smaller than the time interval range considered in the evaluation of *W*. The source of correlation are segments that are shared by the two sequences and are strong enough to overcome the noisy background. The window width *W* is indeed an integration time: increasing it does not affect signal components but progressively lowers noisy contributions. From this point of view, *W* is inversely proportional to a threshold value of the signal-to-noise ratio (SNR) at which the link becomes observable. Two nodes having maximum connectivity so that their sequences are identical would correspond to a vanishing time scale of observability *W*. On the other hand, two unconnected nodes would produce an infinite *W*. Figure 2 shows the *p* value diagram *p*(*k,w*) and the resulting time scale of observability *W* in the case of two nodes that do not exhibit a link and in the case of two nodes exhibiting a link with time scale *W* = 15 s. The time scale of observability *W*, or better its reciprocal, can be considered as a measure of the connectivity strength.

Given a number *N* of nodes within the brain, the number of possible node pairs, and thus of links, is *N*(*N* − 1)/2: the analysis described above is carried on each of the possible pairs. While the results, for example in terms of an adjacency matrix, can be used as a starting point for investigating possible network structures (Gastner and Newman, 2006; Barthélemy, 2011; Perinelli et al., 2018), the goal of the present work is the assessment of the dependence of connectivity, quantified by means of *W*, on the geometric distance between nodes, as discussed below.

For each of the 100 available subjects, 2,556 node pairs are present. For each pair, the link length *d*, i.e., the Euclidean distance between the nodes, was computed out of the corresponding MNI coordinates (Glasser et al., 2016). Given a pair of nodes, the related *d* differs among subjects due to anatomical variability. Consequently, the whole set of 100 subjects results in ~250,000 values of *d* within the range from 5 to 175 mm. For each subject and pair of nodes, the time scale of observability *W* of the corresponding link was assessed out of the related pair of time series. A number of about 150,000 assessments were discarded from further analysis because they did not deliver a finite value of *W*. The described skimming process could be “less severe” by both enhancing the *p*-value threshold to assess cross-correlation and reducing the efficiency threshold. However, any such action would imply a larger number of spurious correlations to be identified as actual links. On the other hand, an infinite value of *W* does not rule out the possibility of a real connection between the related nodes. Increasing the upper limit of time scale *W* that can be assessed, however, would require longer sequences. The analysis was therefore carried out on the remaining set of observable links, having a size of ~100,000.

### 2.3. Subject Dependence of Relative Link Occurrence

**Figure 3:**
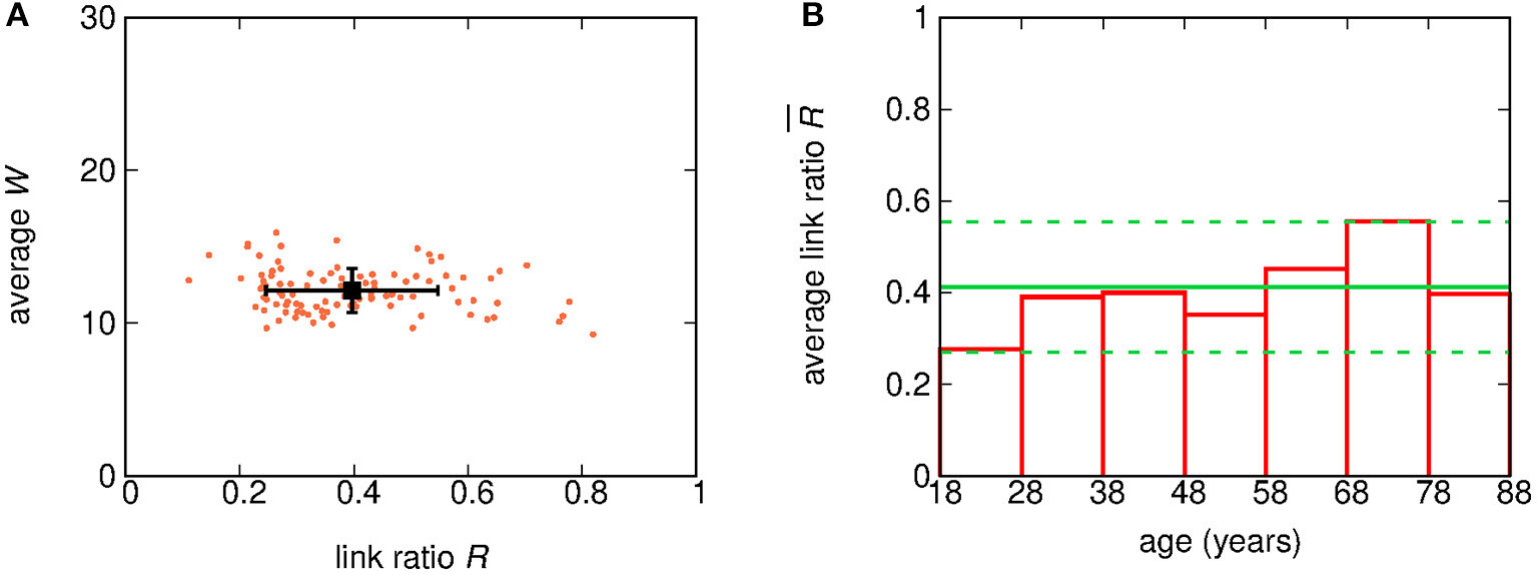
**(A)** Scatter plot of the ratio *R* and the average *W* for each of the 100 subjects. Each point corresponds to one subject. The coordinates of the black square dot correspond to the average *R* and *W* over all subjects, while errorbars show the related standard deviations. **(B)** Bar plot of the average $\bar{R}$ of the ratio *R* for subjects grouped by age decade. The green solid line shows the grand-mean value of $\bar{R}$, while green dashed lines bound the 95% (2 σ) confidence region for $\bar{R}$.

In order to check the variability of the percentage of observable links among subjects, we computed for each subject the ratio *R* of the number of detected links and the number of available pairs of nodes. Figure 3A shows a scatter plot of the ratio *R* and the average *W* for each subject. Moreover, the subject-wise averages of both *R* and *W*, as well as the related standard deviations, are also shown. The clustering observed in the scatter plot of Figure 3A suggests that our results exhibit little subject-to-subject variability.

In addition, in order to highlight any age-related variability, the value of *R* was averaged over subjects grouped by age. Figure 3B shows the average value of *R* for sets of subjects grouped by decades (18–27, 28–37 years, …). The average value of $\bar{R}$ turns out to be (0.40 ± 0.09). While two decades, namely 18−28 yrs and 68−78 yrs, show fluctuations larger than the standard error, there is no clear hint of a systematic age-related variability.

### 2.4. Functional Relationship Between *W* and *d*

**Figure 4:**
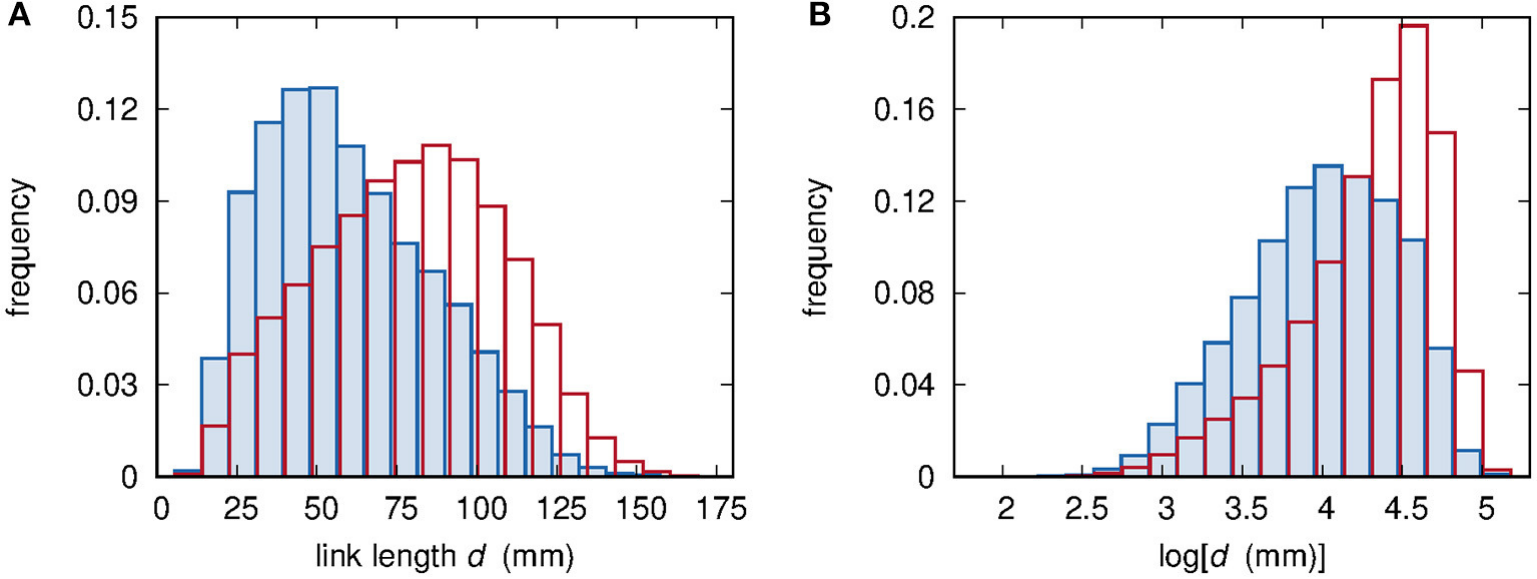
**(A)** Histograms of the ~250,000 available values of link length *d* (red lines) and of the ~100,000 values of *d* corresponding to observable links (blue lines and shaded areas). **(B)** Histograms of the values of log[*d*(mm)]; color meaning is the same as in **(A)**.

As a preliminary analysis step, the sample distributions of *d* and *W* are considered. Figure 4A shows the sample distribution of link lengths *d*. Two histograms are reported: the first one refers to the whole set of available distances, while the second one includes only observable links, i.e., node pairs for which the assessment of *W* provided a finite value. The histograms of Figure 4B are instead built by evaluating the logarithm of distances. The sample distribution of *d* evaluated by including only observable links is shifted toward smaller *d* values, thus implying that smaller distances correspond to a higher probability of observing a link.

**Figure 5:**
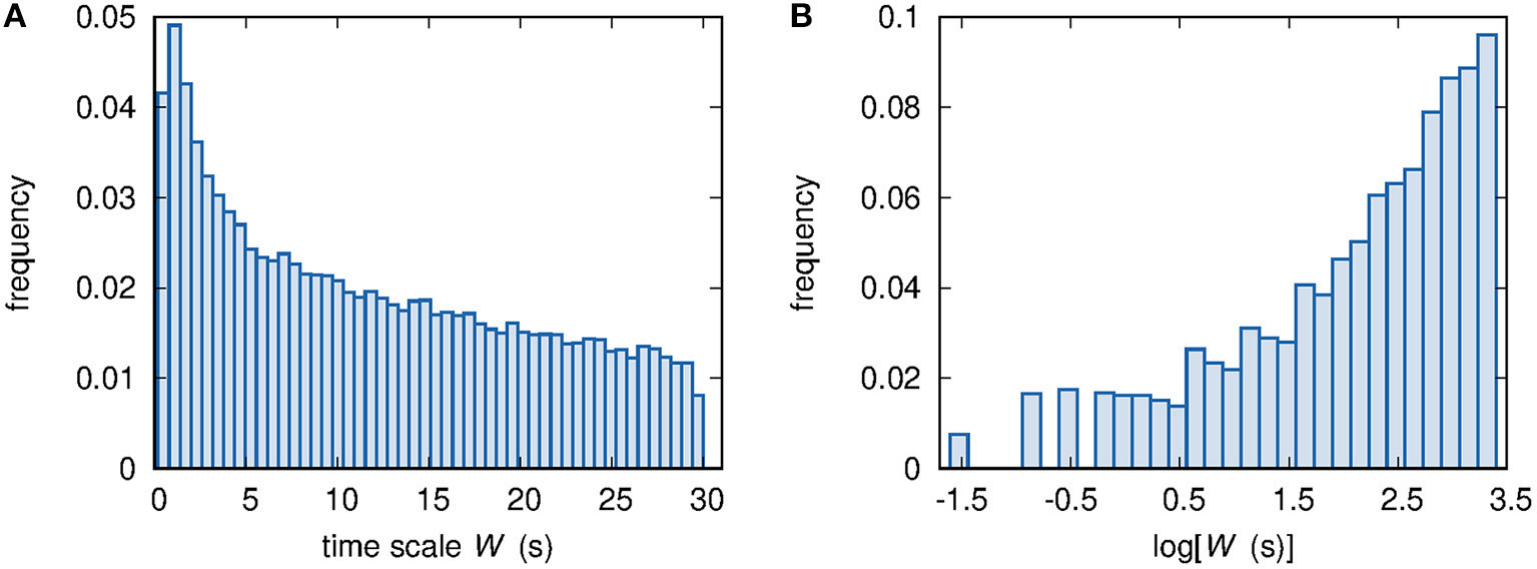
**(A)** Histogram of the ~100,000 available values of time scale *W*. **(B)** Histogram of the ~100,000 available values of log[*W*(s)].

Taking into account observable links only, Figures 5A,B show the histograms of the time scale *W* and its logarithm log[*W*(s)], respectively. The comparison of the histograms shown in Figures 4, 5 prompts us to set some constraints on the form of the mathematical expression of the relationship between *W* and *d*. First, no linear mapping of the abscissa axes can lead to an overlap between the histograms shown in Figures 4A, 5A. Therefore, there is no evidence of a linear relationship between *d* and *W*. Second, a similar argument applied to the histograms of the logarithm of the two variables *d* and *W*, respectively shown in Figures 4B, 5B, rules out a power-law relationship. Finally, exponential or logarithmic relationships are also ruled out because of the impossibility of mapping Figure 4A onto Figure 5B or Figure 4A onto Figure 5A, respectively, via a linear rescaling of the abscissa axes.

**Figure 6:**
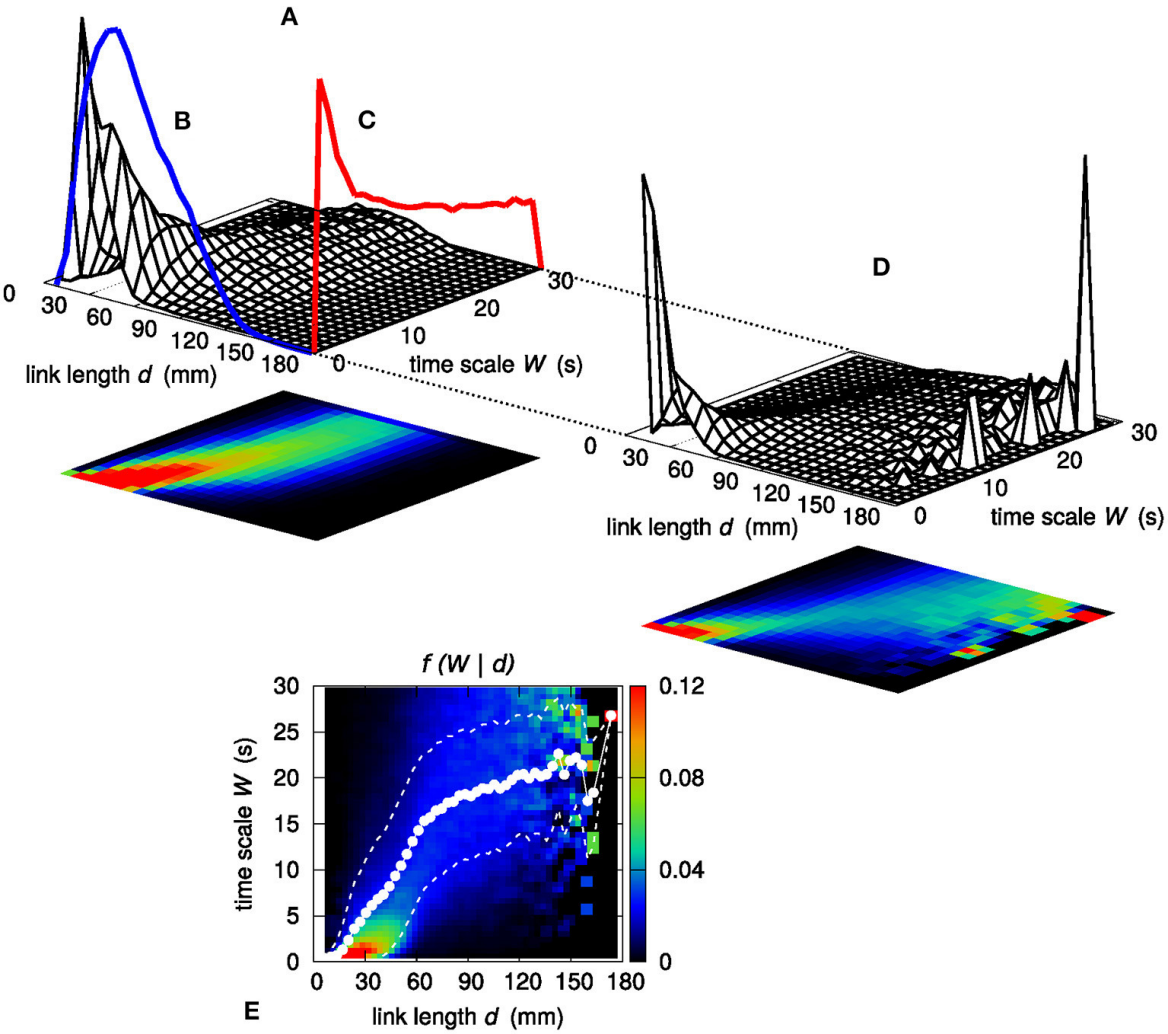
**(A)** Joint sample probability distribution *f* (*d, W*). The surface plot and the corresponding color map projection on the *d, W* plane are obtained by partitioning both the *d* and the *W* range in 25 bins each. Integrating *f* (*d, W*) along *W* yields the sample marginal probability distribution of *d*, which is shown by means of a blue line **(B)**. Similarly, the red line **(C)** shows the marginal sample probability distribution of *W*. **(D)** Sample conditional probability distribution *f* (*W* | *d*) of *W* given *d*, evaluated as *f* (*d, W*)/*g*_*d*_(*d*). **(E)** Color map representation of the sample conditional probability distribution *f* (*W* | *d*); the *d* and the *W* range are partitioned in 50 bins each. White dots show the average value $\bar{W}$ of *W* given *d*. White dashed lines bound the 68% confidence region for *W*, namely the region within 1σ from the average $\bar{W}$.

To further investigate the relationship between link length *d* and time scale of observability *W*, following the approach introduced in Perinelli et al. (2019), the characterization of the dependence of *W* on *d* is carried out by assessing sample conditional probability distributions *f* (*W* | *d*). Figure 6A shows the joint sample probability distribution *f* (*d, W*) obtained by partitioning both the *d* range and the *W* range in 25 bins each. The two sample marginal distributions *g*_*d*_(*d*) and *g*_*W*_(*W*), which are obtained by integrating *f* (*d, W*) along the *W* and *d* axis, respectively, are shown as solid lines in Figures 6B,C. Taking the ratio *f* (*W* | *d*) = *f* (*d, W*)/*g*_*d*_(*d*) yields the sample conditional distribution *f* (*W* | *d*) shown in Figure 6D. For the sake of clarity, the color map representing *f* (*W* | *d*) is also displayed in Figure 6E, in which the number of bins along each direction is increased to 50.

**Figure 7:**
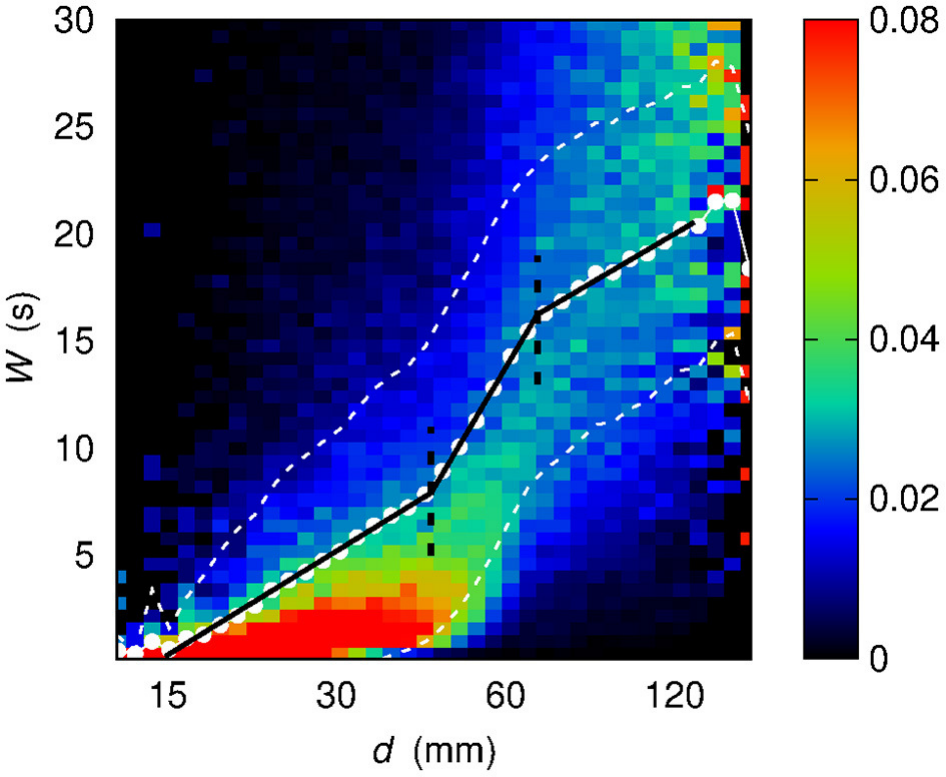
Conditional sample distribution *f* (*W* | log *d*) of *W* given log *d* (the *d* axis is in log scale) obtained by partitioning both the log *d* range and the *W* range in 50 bins. White dots show the average value $\bar{W}$ of *W* given log *d*. White dashed lines bound the 68% confidence region for *W*, namely the region within 1σ from the average $\bar{W}$. Black solid lines stem from a best-fit procedure (see section 3) concerning a piecewise-linear relationship between *W* and log *d*. Black dashed lines correspond to the log *d* values at which the slope of the piecewise-linear relationship changes.

Figure 7 shows the sample conditional probability distribution *f* (*W* | log *d*) obtained by considering the logarithm of distance expressed in millimeters (henceforth, log[*d*(mm)] is simply written as log *d*). As for Figures 6E, 7 also shows the average value of *W*, henceforth referred to as $\bar{W}$, evaluated as a weighted sum:

$$\bar{W}(\log d)=\sum_{\left\{W\;|\;\log(d)\right\}}\;W\;\cdot f(W\;|\;\log d),\;$$

where the sum runs over all *W* bins having the same log *d* (the evaluation of $\bar{W}$ in the linear case of Figure 6E is computed in a similar way). Beside $\bar{W}$, the related standard deviation is also evaluated. In principle, one might consider the median of *W* instead of the sample mean. However, relying on sample means allows to associate an uncertainty—and therefore a confidence interval—to the estimated value $\bar{W}$ by computing the related standard deviation.

While the dependence of $\bar{W}$ on *d* in the linear case shows at least six different slopes, and, in addition, an irregular sequence of transitions from concavity to convexity, the dependence of $\bar{W}$ on log *d* appears to be more regular: the functional shape of $\bar{W}\left(\log d\right)$ evaluated out of the sample conditional distribution *f* (*W* | log *d*) appears to be well-described by a piecewise linear curve, made of three segments. Section 3 describes the results of the assessment of this piecewise-linear curve on different sets of data.

### 2.5. Independence of the Results on the Selected Nodes

In order to test whether the results of the present analysis are independent of the set of randomly chosen nodes, we extracted additional 72 nodes as a control set. This control set was extracted with the same procedure of the original set with the only additional constraint that no nodes of the control set belong to the original one. Upon selecting seven subjects, each randomly chosen within a uniform age distribution, we computed the joint sample probability distribution *f* (*d, W*) both on the original set and on the control set. Thereupon we implemented a 2-dimensional Kolmogorov-Smirnov test (Press et al., 1997) to test the null hypothesis that the two sample distributions *f* (*d, W*) are mutually compatible: for 6 of the 7 subjects, the resulting *p* value was above 5%, while for a single subject the *p* value was 2%. This subject can be considered as an *expected outlier* by virtue of a Bonferroni corrected significance level of 0.05/7 ≈ 0.01.

The compatibility between the results of the original set of nodes and of the control one suggests that an increased number of nodes does not lead to significant changes in the outcomes of the analysis discussed in the present work. On the other hand, selecting too a smaller set of nodes would unreliably sample the *f* (*d, W*) distribution.

## 3. Results

Three distinct regimes are revealed by representing $\bar{W}$ as a function of log *d*. The regimes are henceforth defined as *d* ⩽ *d*_12_ (first regime), *d*_12_ < *d* ⩽ *d*_23_ (second regime), and *d* > *d*_23_ (third regime). As introduced in section 2.4, the sample conditional distribution *f* (*W* | log *d*) is here described by means of a piecewise linear curve. It is worth mentioning that alternative functional forms can be considered as candidates to describe the observed dependence of time scale on distance. However, a piecewise linear relationship turns out to adequately capture the three-regimes behavior while relying on as few as six parameters, four describing the offset and the three slopes and two describing the boundaries of the three regions.

To identify and characterize these regimes, a two-steps best-fit procedure was implemented as follows. First, upon a manual setting of *d*_12_ and *d*_23_, three straight lines are fit over the three resulting ranges. The two abscissae of intersection between these straight lines, which are highlighted in Figure 7 by means of green dashed lines, are taken as the new estimates of *d*_12_ and *d*_23_, thus correcting the preliminary manual settings. Finally, on the three improved ranges stemming from the previous step, a second fit procedure is carried out to assess the final values of the slope of the three straight lines. The outcome of this two-steps procedure is a piecewise-linear best-fit curve.

In Figure 7 the piecewise-linear curve fitted on the whole set of data is shown by means of a black solid line. The slope-changing abscissa values turn out to be *d*_12_ = (44 ± 2) mm and *d*_23_ = (68.3 ± 0.9) mm. The slopes of the three regimes are *m*_1_ = (7.0 ± 0.1) s, *m*_2_ = (19.1 ± 0.5) s, *m*_3_ = (6.7 ± 0.5) s.

**Figure 8:**
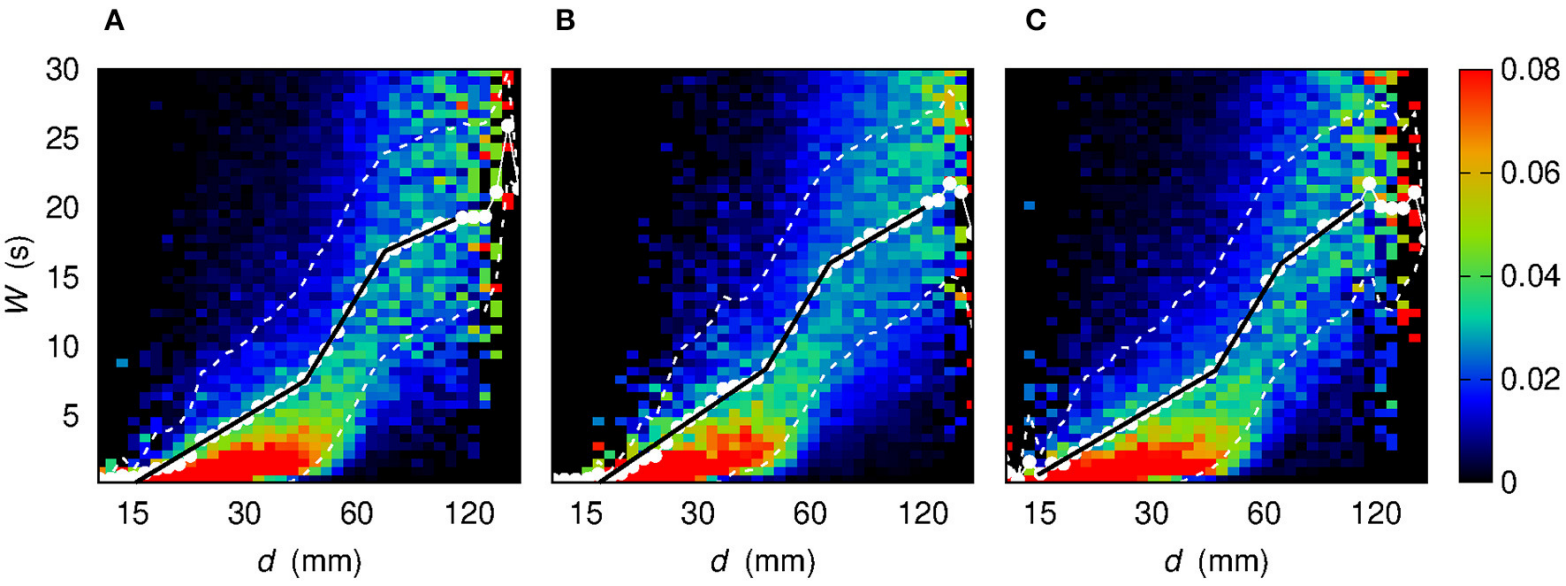
Conditional sample probability distribution *f* (*W* | log *d*) (the *d* axis is in log scale) obtained by partitioning both the log *d* range and the *W* range in 50 bins. Each color map corresponds to a different sets of links. **(A)** Links for which both nodes are located in the left hemisphere. **(B)** Links for which the two nodes are located in opposite hemispheres. **(C)** Links for which both nodes are located in the right hemisphere. White dots show the average value $\bar{W}$ of *W* given log *d*. White dashed lines bound the 68% confidence region for *W*, namely the region within 1σ from the average $\bar{W}$. Black solid lines stem from a best-fit procedure to assess the piecewise-linear relationship between *W* and log *d*.

The results presented above concern the whole set of observable links and subjects and are reported also in Table 2. However, one might wonder whether these outcomes change if the analysis is carried out by considering inter-hemisphere or intra-hemisphere links. We therefore investigated three disjoint sets: a first set containing links for which both nodes are located in the left hemisphere; a second set made of links for which each node belongs to an opposite hemisphere; a third set made of links for which both nodes are located in the right hemisphere. Figure 8 shows the resulting maps of the sample conditional probability distribution *f* (*W* | log *d*) for these three sets of links. Again, three regimes, corresponding to three slopes of a linear $\bar{W}\left(\log d\right)$ relationship, can be identified. The characterization of the three regimes, in terms of best-fit parameters, leads to the three slopes and the two slope-changing abscissa values that are also reported in Table 2.

**Table 2:** Results of the characterization of the piecewise-linear relationship for four different conditions.

**Hemispheres**	***m*_1_ (s)**	***m*_2_ (s)**	***m*_3_ (s)**	***d*_12_ (mm)**	***d*_23_ (mm)**
All links	7.0 ± 0.1	19.1 ± 0.5	6.7 ± 0.5	44 ± 2	68.3 ± 0.9
Left-Left	7.0 ± 0.2	19.0 ± 0.5	5.3 ± 0.7	44 ± 4	71 ± 1
Left-Right	8.0 ± 0.4	19.4 ± 0.1	6.9 ± 0.5	46 ± 8	67.5 ± 0.8
Right-Right	6.9 ± 0.1	19.1 ± 0.7	8.8 ± 0.5	44 ± 2	67 ± 1

*Parameters m_1_, m_2_, m_3_ correspond to the slopes of the first, second, and third linear regimes, respectively, while d_12_, d_23_ are the slope-changing abscissa values*.

The outcomes of the analysis provide a robust evidence of the existence of three distinct regimes of *W* as a function of *d*. Consequently, the three-regime separation turns out to be a shared property of all kinds of links, either concerning nodes belonging to the same hemisphere (intra-hemisphere) or to opposite hemispheres (inter-hemisphere).

More in detail, the slope-changing abscissa values *d*_12_ and *d*_23_ are mutually compatible among the four sets of links listed in Table 2. The same occurs for the first two slopes *m*_1_, *m*_2_, a fact that might be interpreted in terms of hemisphere-independent processes that govern network topology for “small” and “intermediate” distances. The remaining slope *m*_3_ exhibits a higher variability: the larger slope in the Right-Right case with respect to the Left-Right and Left-Left ones might be due to a different mechanism underlying link formation. The fact that *m*_3_ < *m*_2_ can be also due to saturation, namely to the geometric boundedness of brain.

### 3.1. Comparison With Previous Assessments

**Figure 9:**
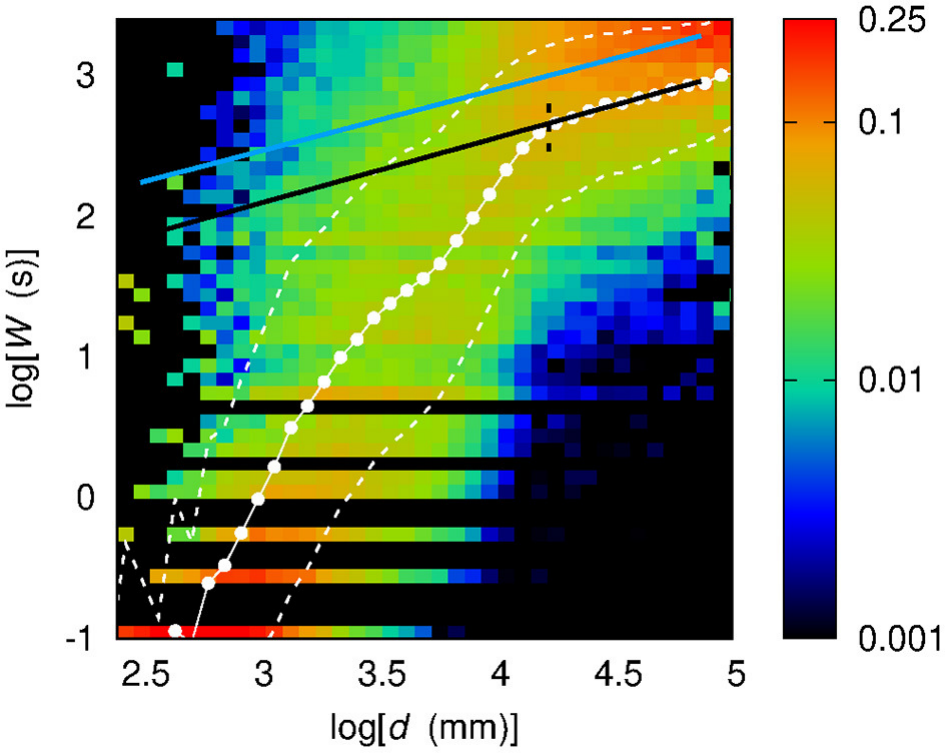
Conditional sample distribution *f*(log *W* |log *d*) of log *W* given log *d* obtained by partitioning both the log *d* range and the log *W* range in 50 bins. White dots show the average value $\bar{\log W}$ of log *W* given log *d*. White dashed lines bound the 68% confidence region for log *W*, namely the region within 1σ from the average $\bar{\log\left(W\right)}$. The black dashed line corresponds to the log *d* value marking the separation between the second and third regimes. The black solid line stems from a best-fit procedure of the same power-law equation on points belonging to the third regime. The light blue solid line shows the results of the analysis from a previous work (Perinelli et al., 2019) concerning a power-law relationship between *W* and *d*.

A previous paper by our group (Perinelli et al., 2019) described a similar approach and suggested a power-law relationship between *W* and *d*, which can be expressed as $W=W_{0}{\left(\frac{d}{d_{0}}\right)}^{\gamma }$. Upon setting *d*_0_ = 75 mm, a best-fit procedure resulted in γ = (0.44 ± 0.1) and *W*_0_ = (20.9 ± 0.2) s. This equation is highlighted in Figure 9, which also shows the sample conditional probability distribution *f* (*W* | *d*) of the dataset used in the present work. It is worth noticing that, while the curve is shifted with respect to the values of $\bar{W}$, the curve slope for large values of *d* is approximately the same. This was confirmed by performing a best-fit procedure with the equation from Perinelli et al. (2019) on the points belonging to the third regime *d* > *d*_23_. The fit resulted in a value of γ = (0.46 ± 0.02), which is compatible with the previous results. The reason for the different behavior in the case of the points corresponding to smaller distances could be due to the improved preprocessing techniques implemented in the present work, particularly in terms of higher SNR. As a result, a greater number of links at lower time scales *W* was detected, which allowed to increase the resolution in the low *W*, and consequently, in the low *d* regime.

## 4. Conclusions

In this paper, we investigated the functional relation between brain connectivity and geometric distance. We used MEG recordings from 100 healthy subjects in resting state to reconstruct time series of cortical activity in 72 randomly chosen nodes. For all possible pairs of nodes, besides the Euclidean distance, we assessed, if any, the degree of connectivity between the nodes by relying on a novel method based on zero-delay cross-correlation. The relationship between geometric distance and connectivity was then analyzed by inferring joint and conditional sample probability distributions. The analysis was also performed separately on inter-hemispheric pairs and intra-hemispheric pairs.

While previous works hinted at a power-law relationship between distance and connectivity, our results suggest that distances can be distinguished in three regimes and that, in each regime, connectivity depends on the logarithm of distance. A logarithmic dependence might hint at the involvement of information-related mechanisms, while the multiple regimes can be related to small-world modular network architecture supporting integrated/segregated processing.

The results presented in the present work can be improved by taking into account more detailed definitions of distance. In this framework one could rely, for example, on diffusion imaging or g-ratio methods to assess physical connections between nodes. This possibility, though not in the scope of the present manuscript, makes up an interesting future development.

## Data Availability Statement

The dataset used for this study is publicly available at http://www.mrc-cbu.cam.ac.uk/datasets/camcan/. Data collection and sharing for this project was provided by the Cambridge Centre for Ageing and Neuroscience (CamCAN). Analysis code for data pre-processing and preparation is publicly available on Open Science Framework (10.17605/OSF.IO/94WJ3) under the GNU General Public License (GPL) 3.0. Code used for cross-correlation analysis is part of the open source *NetOnZeroDXC* package available at https://github.com/LeonardoRicci/netOnZeroDXC.

## Ethics Statement

The studies involving human participants were reviewed and approved by data collection was conducted in compliance with the Helsinki Declaration and was approved by the University of Cambridge ethics committee (Cambridgeshire 2 Research Ethics Committee—reference: 10/H0308/50). The patients/participants provided their written informed consent to participate in this study.

## Author Contributions

DT pre-processed the dataset and including source reconstruction. LR selected the analytical techniques. MC and AP analyzed the data with inputs from DT. All authors discussed and interpreted the results, and wrote the manuscript.

## Conflict of Interest

The authors declare that the research was conducted in the absence of any commercial or financial relationships that could be construed as a potential conflict of interest.

## Footnotes

^1^See http://www.neuro.uni-jena.de/cat/ for the CAT toolbox (accessed June 2020).
